# Changes in Life Expectancy Between 2019 and 2020 in the US and 21 Peer Countries

**DOI:** 10.1001/jamanetworkopen.2022.7067

**Published:** 2022-04-13

**Authors:** Steven H. Woolf, Ryan K. Masters, Laudan Y. Aron

**Affiliations:** 1 Center on Society and Health, Virginia Commonwealth University School of Medicine, Richmond; 2 Department of Sociology, University of Colorado Boulder, Boulder; 3 Health Policy Center, Urban Institute, Washington, DC

## Abstract

**Importance:**

Prior studies reported that US life expectancy decreased considerably in 2020 because of the COVID-19 pandemic, with estimates suggesting that the decreases were much larger among Hispanic and non-Hispanic Black populations than non-Hispanic White populations. Studies based on provisional data suggested that other high-income countries did not experience the large decrease in life expectancy observed in the US; this study sought to confirm these findings according to official death counts and to broaden the pool of comparison countries.

**Objective:**

To calculate changes in US life expectancy between 2019 and 2020 by sex, race, and ethnicity and to compare those outcomes with changes in other high-income countries.

**Design, Setting, and Participants:**

This cross-sectional study involved a simulation of life tables based on national death and population counts for the US and 21 other high-income countries in 2019 and 2020, by sex, including an analysis of US outcomes by race and ethnicity. Data were analyzed in January 2022.

**Exposures:**

Official death counts from the US and 21 peer countries.

**Main Outcomes and Measures:**

Life expectancy at birth and credible range (CR) based on 10% uncertainty.

**Results:**

Between 2019 and 2020, US life expectancy decreased by a mean of 1.87 years (CR, 1.70-2.03 years), with much larger decreases occurring in the Hispanic (3.70 years; CR, 3.53-3.87 years) and non-Hispanic Black (3.22 years; CR, 3.03-3.40 years) populations than in the non-Hispanic White population (1.38 years; CR, 1.21-1.54 years). The mean decrease in life expectancy among peer countries was 0.58 years (CR, 0.42-0.73 year) across all 21 countries. No peer country experienced decreases as large as those seen in the US.

**Conclusions and Relevance:**

Official death counts confirm that US life expectancy decreased between 2019 and 2020 on a scale not seen in 21 peer countries, substantially widening the preexisting gap in life expectancy between the US and peer countries. The decrease in US life expectancy was experienced disproportionately by Hispanic and non-Hispanic Black populations, consistent with a larger history of racial and ethnic health inequities resulting from policies of exclusion and systemic racism. Policies to address the systemic causes of the US health disadvantage relative to peer countries and persistent racial and ethnic inequities are essential.

## Key Points

**Question:**

How did US life expectancy change between 2019 and 2020, and how did that compare with changes in life expectancy in 21 other high-income countries?

**Findings:**

In this cross-sectional study, calculations of life expectancy based on official death counts revealed that US life expectancy decreased by 1.87 years overall, and by 3.70 years in Hispanic populations and 3.22 years in non-Hispanic Black populations. The decrease in life expectancy in peer countries was a mean of 0.58 years, with no country experiencing a decrease rivaling that of the US.

**Meaning:**

The large and highly racialized decreases in US life expectancy underscore the growing US health disadvantage relative to peer countries and the need for policies that prioritize health and equity.

## Teaser

This cross-sectional study examines death counts in the US and 21 peer countries between 2019 and 2020 to evaluate life expectancy disparities between the US and peer countries and racial disparities within the US.

## Introduction

In 2020, the US documented more deaths from COVID-19 than any other country and, even after adjusting for population size, had among the highest COVID-19 mortality rates.^1^ The pandemic caused deaths from COVID-19 and an increase in non-COVID deaths resulting from disruptions produced by the pandemic. Studies of excess deaths (ie, deaths from all causes in excess of the number that would be projected under normal circumstances) have shown that the US rate of excess deaths was among the highest in the world.^2^

Life expectancy, a measure that summarizes a population’s mortality rates in a given year, is commonly used to track mortality trends over time or to compare mortality profiles across multiple countries. Life expectancy, a term that is often misunderstood, reflects how long a group of people can expect to live were they to experience at each age the prevailing age-specific mortality rates of that year. The high prevailing mortality rates in 2020, reflecting the first year of the global COVID-19 pandemic, led to large decreases in life expectancy in many countries, including the US. According to the National Center for Health Statistics (NCHS), US life expectancy decreased by 1.8 years in 2020.^3^

Given that US COVID-19 mortality and excess deaths were among the highest in the world, the decline in US life expectancy likely exceeded declines in other countries, but research confirming this hypothesis is limited. A larger decrease in life expectancy in the US than in other countries would be important because the country entered the pandemic with the lowest life expectancy among high-income countries, an ignominious status it has held for decades.^4^ Since 2010, while life expectancy in other countries continued to increase, US life expectancy has remained stagnant and decreased for 3 consecutive years in 2014 to 2017, widening the life expectancy gap with peer nations.^5^

Preliminary evidence for 2020 suggests that the gap with other countries widened much further during the COVID-19 pandemic, reflecting the large number of US deaths. A 2021 study,^6^ which relied on provisional US death counts, estimated that US life expectancy decreased by 1.87 years between 2018 and 2020, compared with a mean decrease of 0.22 years in 16 high-income countries. An alarming finding of that study was the highly racialized nature of the decrease in US life expectancy, which plummeted by 3.88 years and 3.25 years, respectively, in Hispanic and non-Hispanic Black populations, compared with 1.36 years in the non-Hispanic White population.^6^ An international study^7^ that included the US along with 36 other countries also reported a large decrease in US life expectancy but relied on modeling to project death counts and did not examine US outcomes by race and ethnicity.

To our knowledge, this modeling study is the first to use recently released official US death counts for 2020, rather than provisional data, to calculate changes in US life expectancy between 2019 and 2020 by sex, race, and ethnicity and to compare those outcomes with changes in 21 other high-income countries.

## Methods

Because the study did not involve human participants, it was exempt from institutional review under 45 CFR §46.101(b)(4). The study followed the Strengthening the Reporting of Observational Studies in Epidemiology (STROBE) reporting guideline.

### US Life Expectancy Calculations

US life expectancy was calculated for the total population, by sex, race, and ethnicity. US data were examined for 3 racial and ethnic groups that constitute more than 90% of the total population: Hispanic, non-Hispanic Black, and non-Hispanic White populations. Although many US individuals self-identify as Latino or Latina, we use the term Hispanic to maintain consistency with data sources.^8^ Estimates for other important racial groups, such as Asian, Pacific Islander, and American Indians and Alaska Natives, could not be calculated because NCHS does not provide the required mortality data to generate life tables for these populations.

Life tables for US populations in 2019 and 2020 were generated in 5 steps. First, official US life tables for 2018 were obtained for the total US population (1 life table); female and male populations (2 tables); Hispanic, non-Hispanic White, and non-Hispanic Black populations (3 tables); and the female and male population in each racial and ethnic group (6 tables).^9^ These 12 tables were each converted into 5-year abridged life tables composed of 22 age groups (0 to <1 years, 1 to <5 years, 5 to <10 years, ..., 95 to 100 years, and ≥100 years). These abridged life tables were used to calculate 2018 period age-specific death rates, m_x_, for each population.^10^

Second, age-specific mortality rates for the 12 populations were calculated for 2018, 2019, and 2020 from official death counts provided for 19 age groups (0 to <1 years, 1 to <5 years, 5 to <10 years, …, 80 to 85 years, and ≥85 years) in the NCHS Restricted-Access Detailed-Mortality–Limited Geography Files^11^ (numerator) and mid-year (July 1) population counts for 2018, 2019, and 2020 provided by the US Census Bureau's Vintage 2020 estimates in the US Centers for Disease Control and Prevention WONDER Online Database (denominator).^12^ Third, this information was used to calculate age-specific mortality rate ratios (RRs) for 2019 and 2020 relative to 2018. Fourth, those RRs were multiplied by m_x_ in official US life tables for 2018 to estimate m_x_ for 2019 and 2020.^9^ Finally, the estimated m_x_ values for 2019 and 2020 were used to generate life tables for 2019 and 2020. This approach, which has been validated and tested previously,^6^ is described in detail in the eAppendix and eTable in the Supplement.

### Peer Country Life Expectancy Calculations

Countries were included in the peer comparison group if they were high-income advanced democracies and had available data sources for calculations. The 21-country peer comparison group included Austria, Belgium, Canada, Denmark, England and Wales (combined), Finland, France, Germany, Israel, Italy, Netherlands, New Zealand, Northern Ireland, Norway, Portugal, Scotland, South Korea, Spain, Sweden, Switzerland, and Taiwan.

Five-year abridged life tables for each country’s female and male populations in 2018 were obtained from the Human Mortality Database (HMD).^13^ Direct sources were used for Germany, Israel, and New Zealand because the HMD did not provide 2018 life tables for these countries. To calculate each country’s female and male age-specific death rates in 2018, 2019, and 2020, weekly age-specific death counts in each country’s female and male population were obtained for 2018, 2019, and 2020 from the January 17, 2022 release of the HMD Short-term Mortality Fluctuation original source files,^14^ and were merged with age-specific estimates of each country’s male and female populations in 2018, 2019, and 2020. Population estimates were obtained from each country's central statistical agency (see eAppendix in Supplement for hyperlinks to original sources).

Female and male life expectancies for 2019 in Austria, Denmark, Netherlands, Norway, Portugal, Sweden, and Taiwan were obtained from 2019 HMD life tables, and male and female life expectancies in South Korea were obtained from original sources.^15^ For all other peer countries, 2019 male and female life expectancies were modeled using m_x_ values from the 2018 HMD life tables and applying the 2019 to 2018 age-specific RRs derived from the HMD-Short-term Mortality Fluctuation data. The same procedure, using 2020 to 2018 RRs, was used to model 2020 life expectancy estimates for all peer countries.

### Statistical Analysis

This approach to estimating life expectancy could introduce error, both because the US estimates relied on estimates of 5-year death rates for 2020 and an open-ended 85 years or older age interval, and because estimates for peer countries relied on provisional death counts. Calculating confidence intervals would be inappropriate in this circumstance. For example, traditional calculations would generate wide confidence intervals for countries with smaller populations, even though such countries (eg, Denmark) often produce more trustworthy death counts than large countries such as the US. Instead, a credible range (CR) was estimated for each country’s life expectancy by adding 10% random uncertainty to age-specific mortality risks and then using Python statistical software version 3.9.1 (Python Software Foundation) to simulate 50 000 life tables for the country’s 2020 population by sex (and by race and ethnicity for US estimates). Adding 10% uncertainty was chosen over alternative ranges (eg, 5% or 15%) because it most closely replicated official age-specific mortality rates reported by NCHS,^3,16,17^ the HMD,^13^ and international investigators.^7^ Data were analyzed in January 2022.

## Results

### United States

Between 2019 and 2020, US life expectancy decreased by a median of 1.87 years (CR, 1.70-2.03 years), from 78.86 years to 76.99 years (Table). The decrease in life expectancy was larger among the male population (2.13 years; CR, 1.96-2.30) than the female population (1.51 years; CR, 1.35-1.67).

**Table.** Life Expectancy in the US and 21 Peer Countries, 2019 and 2020

Country	Life expectancy in 2019 and estimates for 2020, median (credible range), y^a^					
	Total population		Female population		Male population	
	2019	2020	2019	2020	2019	2020
Peer countries						
Austria	81.91	81.14 (80.99 - 81.30)	84.20	83.52 (83.37 - 83.67)	79.54	78.73 (78.57 - 78.89)
Belgium	81.84	80.70 (80.55 - 80.86)	84.02	82.96 (82.81 - 83.11)	79.60	78.43 (78.28 - 78.59)
Canada	82.32	81.40 (81.24 - 81.56)	84.34	83.44 (83.29 - 83.60)	80.24	79.36 (79.20 - 79.52)
Denmark	81.43	81.40 (81.24 - 81.56)	83.42	83.35 (83.19 - 83.50)	79.44	79.45 (79.29 - 79.61)
England and Wales	81.71	80.46 (80.31 - 80.62)	83.53	82.46 (82.31 - 82.62)	79.85	78.47 (78.32 - 78.64)
Finland	81.91	81.80 (81.64 - 81.96)	84.53	84.56 (84.42 - 84.72)	79.22	79.05 (78.89 - 79.21)
France	82.76	82.07 (81.92 - 82.23)	85.63	85.06 (84.91 - 85.21)	79.76	79.01 (78.85 - 79.17)
Germany	81.16	80.77 (80.62 - 80.93)	83.67	83.37 (83.22 - 83.52)	78.94	78.48 (78.32 - 78.64)
Israel	82.40	82.02 (81.87 - 82.18)	84.31	84.18 (84.03 - 84.33)	80.64	80.05 (79.88 - 80.21)
Italy	83.34	82.16 (82.00 - 82.31)	85.40	84.43 (84.28 - 84.58)	81.14	79.83 (79.67 - 79.98)
Netherlands	82.05	81.39 (81.24 - 81.54)	83.56	82.87 (82.73 - 83.02)	80.46	79.84 (79.69 - 79.99)
New Zealand	81.61	82.25 (82.11 - 82.39)	83.56	84.09 (83.96 - 84.23)	80.00	80.65 (80.50 - 80.80)
Northern Ireland	80.96	79.83 (79.67 - 79.99)	82.74	81.72 (81.56 - 81.88)	79.02	77.91 (77.75 - 78.07)
Norway	82.96	83.03 (82.88 - 83.18)	84.70	84.72 (84.57 - 84.87)	81.18	81.32 (81.17 - 81.47)
Portugal	81.71	80.80 (80.65 - 80.96)	84.56	83.78 (83.64 - 83.93)	78.64	77.66 (77.50 - 77.82)
Scotland	79.29	78.33 (78.17 - 78.49)	81.26	80.60 (80.45 - 80.76)	77.28	76.06 (75.90 - 76.23)
South Korea	83.29	83.53 (83.37 - 83.68)	86.30	86.34 (86.19 - 86.49)	80.27	80.43 (80.27 - 80.59)
Spain	83.56	82.13 (81.97 - 82.28)	86.21	84.87 (84.73 - 85.02)	80.83	79.39 (79.23 - 79.55)
Sweden	83.06	82.26 (82.11 - 82.41)	84.73	84.12 (83.98 - 84.27)	81.35	80.41 (80.26 - 80.57)
Switzerland	83.79	82.95 (82.80 - 83.10)	85.58	84.97 (84.83 - 85.12)	81.89	80.87 (80.72 - 81.02)
Taiwan	80.58	81.11 (80.94 - 81.27)	83.78	84.26 (84.11 - 84.42)	77.53	78.07 (77.90 - 78.25)
Peer mean	82.08	81.50 (81.35 - 81.66)	84.29	83.79 (83.65 - 83.95)	79.85	79.21 (79.05-79.37)
US						
Total	78.86	76.99 (76.83 - 77.16)	81.39	79.88 (79.72 - 80.04)	76.32	74.19 (74.02 - 74.36)
Hispanic^b^	81.86	78.16 (77.99 - 78.33)	84.40	81.58 (81.42 - 81.74)	79.08	74.77 (74.59 - 74.94)
Non-Hispanic Black^b^	74.76	71.54 (71.36 - 71.73)	78.08	75.37 (75.19 - 75.55)	71.30	67.76 (67.56 - 67.95)
Non-Hispanic White^b^	78.78	77.40 (77.24 - 77.57)	81.26	80.10 (79.94 - 80.26)	76.33	74.80 (74.63 - 74.97)

^a^ The credible range of uncertainty for estimates of 2020 life expectancy is bounded by the 5th and 95th percentiles from the distribution of 50 000 simulated life expectancies (see methods and eAppendix in Supplement for details).

^b^ Race and ethnicity were defined by the US Census Bureau and National Center for Health Statistics.

Declines in US life expectancy varied greatly by race and ethnicity: the non-Hispanic Black population experienced a decrease of 3.22 years (CR, 3.03-3.40 years), from 74.76 years in 2019 to 71.54 years in 2020. In contrast, life expectancy in the non-Hispanic White population decreased by 1.38 years (CR, 1.21-1.54 years). Life expectancy in the Hispanic population, which has historically experienced better survival rates than the non-Hispanic White population,^18^ experienced the largest life expectancy decline in 2020, a decrease of 3.70 years (CR, 3.53-3.87 years), from 81.86 years to 78.16 years. Hispanic and non-Hispanic Black men experienced the largest decreases in life expectancy, including decreases of 4.31 years (CR, 4.14-4.49 years) in Hispanic men and 3.54 years (CR, 3.35-3.74 years) in non-Hispanic Black men, compared with a decrease of 1.53 years (CR, 1.36-1.70 years) in non-Hispanic White men.

### Peer Countries

**Figure 1.**
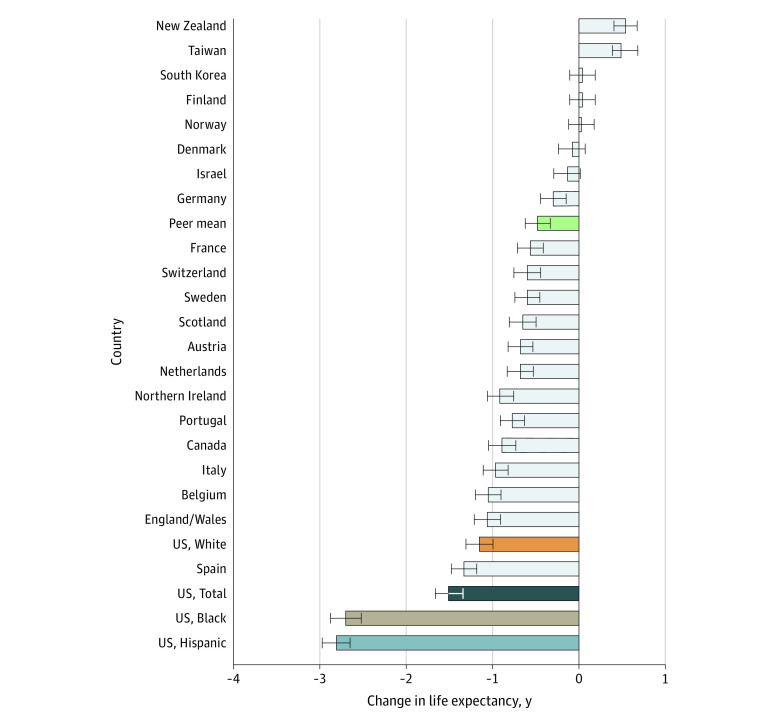
Changes in Female Life Expectancy in the US and 21 Other High-Income Countries Between 2019 and 2020 Horizontal bars represent the credible range (CR) of uncertainty based on model simulations (see methods). The green bar labeled “peer mean” plots the mean for the 21 peer countries.

**Figure 2.**
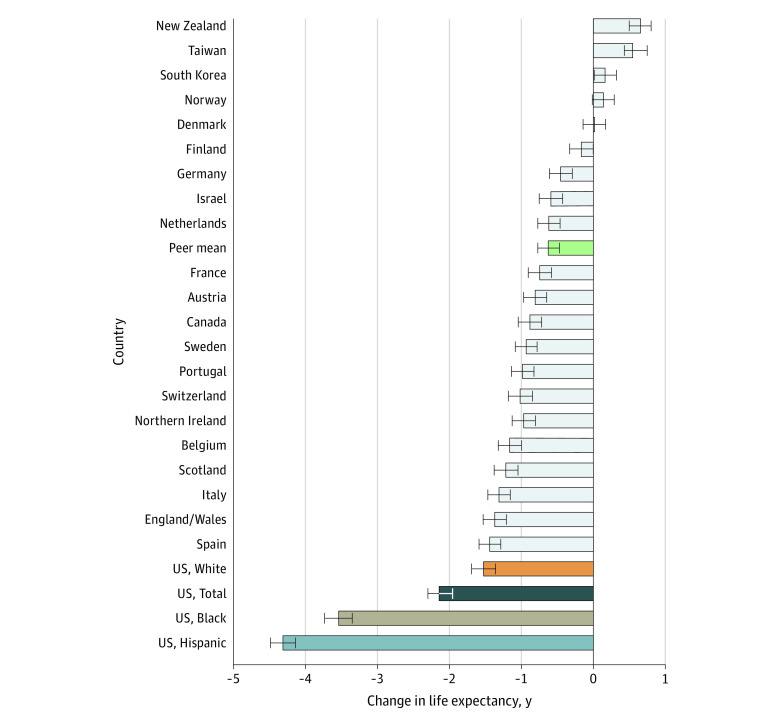
Changes in Male Life Expectancy in the US and 21 Other High-Income Countries Between 2019 and 2020 Horizontal bars represent the credible range (CR) of uncertainty based on model simulations (see methods). The green bar labeled “peer mean” plots the mean for the 21 peer countries.

Across the 21 peer countries, the mean change in life expectancy between 2019 and 2020 was a decrease of 0.58 years (CR, 0.42-0.73 year) (Table). In 2020, mean life expectancy in the peer countries was 81.50 years, 4.51 years higher than US life expectancy (76.99 years). Outcomes in peer countries ranged from a decrease of 1.43 years (CR, 1.28-1.59 years) in Spain to increases in life expectancy in New Zealand, South Korea, and Taiwan. In Denmark, Finland, and Norway, life expectancy did not change significantly (CR included 0). The peer countries that experienced the largest decreases in female life expectancy in 2020 were Spain (1.34 years; CR, 1.19-1.48 years), England and Wales combined (1.07 years; CR, 0.91-1.22 years), and Belgium (1.06 years; CR, 0.91-1.21 years) (Figure 1). The largest decreases in male life expectancy occurred in Spain (1.44 years; CR, 1.28-1.60 years), England and Wales (1.38 years; CR, 1.21-1.53 years), and Italy (1.31 years; CR, 1.16-1.47 years) (Figure 2). No peer country experienced decreases in life expectancy as large as those seen in the US.

## Discussion

Decreases in US life expectancy in 2020 greatly exceeded mean changes in 21 high-income countries, adding to the longstanding and growing life expectancy gap between the US and its peers. In the 1980s, US life expectancy started increasing more slowly than in peer countries, plateauing after 2010.^19^ One analysis, which compared life expectancy for the US and 16 peer countries, estimated that the gap grew from 1.9 years in 2010 to 3.1 years in 2018 and that the COVID-19 pandemic widened the gap to 4.7 years in 2020.^6^ The gap documented in this study, based on the mean of 21 countries, is almost as large at 4.51 years.

The downward trends in US life expectancy since 2010 reflect increasing death rates among young and middle-aged adults,^5^ including higher cause-specific death rates from drug overdoses, cardiometabolic diseases (eg, obesity and diabetes), and other chronic diseases. The pervasiveness of the US health disadvantage, spanning multiple causes of morbidity and mortality, likely has systemic origins. Compared with other high-income countries, the US ranks poorly on social and economic conditions (eg, education, poverty, income inequality, and affordable housing); health-promoting environments and infrastructure (eg, walkability, public transit, and access to healthy foods); social well-being (eg, racial segregation and social isolation); and access to health care and health insurance. In contrast to policies adopted by its peers, US social welfare spending is less equitable and less beneficial to children and families.^20,21,22^ The US also lacks universal health care and provides weaker protections for public health and safety.^4^

Given these systemic vulnerabilities, the US entered the COVID-19 pandemic in a fundamentally weakened state. These preexisting conditions, combined with mismanagement of federal, state, and local pandemic responses and factional public resistance to practices to prevent viral transmission, drove US death rates above those experienced by other countries. The excess deaths included not only those attributed to COVID-19 but also non-COVID deaths associated with social and economic disruptions of the pandemic, along with inadequate or delayed care of acute emergencies and chronic illnesses and behavioral health crises that fueled a record increase in fatal drug overdoses.^23^

Consistent with the long history of racial and socioeconomic health disparities in the US, 2020 deaths were disproportionately borne by Hispanic and non-Hispanic Black people and low-income communities. Life expectancy declined by more than 3 years in the Hispanic and Black populations—more than 4 years among Hispanic men—far exceeding losses in the non-Hispanic White population. The large decreases in life expectancy among Hispanic and Black populations reflect their higher risk of hospitalization and death from COVID-19 and vulnerability to conditions causing non-COVID deaths.^24,25^ The racialized health inequities that were conspicuous in 2020 have existed for generations, the products of systemic racism, segregation, and exclusionary policies.^26^ Historic and current conditions have systematically blocked racial and ethnic minority groups from access to health care, social and economic mobility, and environmental conditions that determine health and life expectancy.^27^

This study’s estimate that US life expectancy decreased by 1.87 years in 2020 is consistent with other reports,^3,6^ which estimated decreases of 1.8 and 1.9 years, respectively. The racialized pattern of these decreases has also been reported previously; provisional data suggested that life expectancy decreased in 2020 by 2 to 3 years and 3 to 4 years, respectively, in the Hispanic and non-Hispanic Black populations and 1 year in the non-Hispanic White population.^28^ This study documents even larger losses of life expectancy in these populations and to our knowledge, is the first to be based on official death counts. Estimates here of life expectancy in peer countries are also consistent with international reports. For example, a 37-country analysis, which estimated that US life expectancy decreased by 2.0 years in 2020, found that none of the 21 countries examined here experienced a decrease greater than 1.4 years.^7^

Until final death counts for 2021 are released, it remains unclear if the large decreases in US life expectancy documented here extended into 2021. However, even with a return to prepandemic mortality rates, the US health disadvantage that has grown over decades will persist without corrective action. US residents will continue to die at higher rates than their counterparts in other advanced democracies, and their health will remain sharply divided along racial and ethnic lines, until the country makes policy choices that optimize health, well-being, and equity.^29^

### Limitations

This study has several limitations. Racial disparities in US outcomes could not be compared with those in peer countries because of inconsistencies in how race and ethnicity are understood and measured across countries. Other limitations included cross-country variation in reporting of deaths, and the exclusion of some high-income countries (eg, Australia and Japan) and US racial groups because of insufficient data. The latter prevented the estimation of life expectancy in US populations believed to have experienced high death rates (eg, American Indians and Alaska Natives).^30^

## Conclusions

In this study, official death counts confirm that US life expectancy decreased between 2019 and 2020 on a scale not seen in 21 peer countries. The decrease in US life expectancy was experienced disproportionately by Hispanic and non-Hispanic Black populations, consistent with a larger history of racial and ethnic health inequities resulting from policies of exclusion and systemic racism. Policies to address the systemic causes of the US health disadvantage relative to peer countries and persistent racial and ethnic inequities are essential.

## Notes

Supplement. **eAppendix.** Supplemental Methods **eTable.** Comparison of Published Estimates and Model Predictions for 2019 Life Expectancy and Changes in Life Expectancy Between 2019 and 2020 in the US, Austria, and Portugal
eReferences
