# Supplementary material for: Changes in Life Expectancy Between 2019 and 2020 in the US and 21 Peer Countries
Source: JAMA Netw Open. 2022 Apr 13;5(4):e227067. doi: 10.1001/jamanetworkopen.2022.7067 (PMC9008499; doi:10.1001/jamanetworkopen.2022.7067)
Supplement: Supplement. — eAppendix. Supplemental Methods eTable. Comparison of Published Estimates and Model Predictions for 2019 Life Expectancy and Changes in Life Expectancy Between 2019 and 2020 in the US, Austria, and Portugal eReferences [file jamanetwopen-e227067-s001.pdf]

Supplemental Online Content

Woolf SH, Masters RK, Aron LY. Changes in life expectancy between 2019 and 2020 in the US and 21 peer countries. *JAMA Netw Open*. 2022;5(4):e227067. doi:10.1001/jamanetworkopen.2022.7067

eAppendix. Supplemental Methods
eTable. Comparison of Published Estimates and Model Predictions for 2019 Life Expectancy and Changes in Life Expectancy Between 2019 and 2020 in the US, Austria, and Portugal
eReferences

This supplemental material has been provided by the authors to give readers additional information about their work.

© 2022 Woolf SH et al. *JAMA Network Open.*

eAppendix. Supplemental Methods

Counts of death among U.S. populations in 2018, 2019, and 2020 were obtained from the National Center for Health Statistics' (NCHS) Restricted-Use Vital Statistics, *2018-2020 Detailed Mortality - Limited Geography* files.[1] These official mortality data are composed of annual counts of death among U.S. populations at each age [0-1), ..., [99-100), 100+ and were collapsed into 19 age intervals [0-1), [1-5), [5-10), ..., [80-85), 85+. Mid-year population estimates in 2018, 2019, and 2020 by sex, race/ethnicity, and 19 age intervals [0-1), [1-5), [5-10), ..., [80-85), 85+ were obtained via the CDC WONDER Online Database from the U.S. Census Bureau file, *Vintage 2020 Estimates*.[2]

Death counts in 2018, 2019, and 2020 by sex and by age in each peer country were obtained from the January 17, 2022 release of the Human Mortality Database (HMD) - Short-term Mortality Fluctuations original input data in standardized format files. The age-specificity of death counts varied across peer countries. For example, 2020 death counts in England/Wales were provided across seven age groups of varying intervals [0-1), [1-15), [15-45), [45-65), [65-75), [75-85), 85+, while 2020 death counts in Norway were provided across 21 five-year age intervals [0-5), [5-10), ..., [95-100), 100+. See the HMD metadata for additional details about each peer country's reports of 2020 deaths. Population counts in 2018, 2019, and 2020 by sex and age were obtained from each country's central statistical agency. Death counts for Canada, the Netherlands, New Zealand, and South Korea were available for five age groups that match the five age groups for the year-specific mortality rates published by the HMD-STMF (i.e., [0-15), [15-65), [65-75), [5-85), 85+). Therefore, we did not combine original death counts with population estimates for these countries, but instead used the mortality rates provided by the HMD-STMF data for five age groups: [0-15), [15-65), [65-75), [75-85), and 85+.

Life expectancy in 2019 for the total, male, and female populations of Austria, Denmark, Finland, Netherlands, Norway, Portugal, Sweden, and Taiwan were obtained from the HMD five-year abridged period life tables. For 2019 life expectancy in South Korea, we used the 2019 life tables generated by KOSIS.

To estimate 2019 and 2020 life tables for each peer country's male and female population, we first estimated period age-specific death rates (m_x) for each country's male and female population in years 2018, 2019, and 2020. We merged estimates of age-specific death counts in the HMD-STMF original input data in standardized format files with the age-specific population counts provided by each country's central statistical agency. Next, we estimated the 2019:2018 and 2020:2018 age-specific mortality rate ratios ($RR_{2019,STMF}$ and $RR_{2020,STMF}$) for each country's total, male, and female populations using the estimated 2018, 2019, and 2020 m_x. Third, to estimate $\boldsymbol{m_{x_{2019}}}$ and $\boldsymbol{m_{x_{2020}}}$ for the 22 age groups used to generate 2019 and 2020 life tables, we multiplied the age-specific $RR_{2019,STMF}$ and $RR_{2020,STMF}$ with the age-specific 2018 m_x reported in each country's HMD 2018 life table:

$$\boldsymbol{m_{x_{2019}}} = m_{2018,HMD} * RR_{2019,STMF} \qquad \boldsymbol{m_{x_{2020}}} = m_{2018,HMD} * RR_{2020,STMF}$$

© 2022 Woolf SH et al. *JAMA Network Open.*

HMD period life tables for Germany, Israel, and New Zealand were not available for 2018. Accordingly, 2018 life tables were obtained directly from the central statistical agencies of Germany, Israel, and New Zealand.

To generate 2019 and 2020 life tables for peer countries, we used $\boldsymbol{m}_{\boldsymbol{x}_{\boldsymbol{2019}}}$ and $\boldsymbol{m}_{\boldsymbol{x}_{\boldsymbol{2020}}}$ and each country's a_x in the 2018 life table to estimate $\boldsymbol{q}_{\boldsymbol{x}_{\boldsymbol{2019}}}$ and $\boldsymbol{q}_{\boldsymbol{x}_{\boldsymbol{2020}}}$ For example, for 2019:

$$\boldsymbol{q}_{\boldsymbol{x}_{\boldsymbol{2019}}} = {(\boldsymbol{m}_{\boldsymbol{x}_{\boldsymbol{2019}}} * n)}\Big/{\left(1 + \left[(n - a_{x_{2018}}) * \boldsymbol{m}_{\boldsymbol{x}_{\boldsymbol{2019}}}\right]\right)}$$

where n is the width of the age interval.

Age-specific death rates for US male and female populations in 2018, 2019, and 2020 were calculated by merging estimates of age-specific counts of death (d_x)[1] for 19 age groups [0-1), [1-5), [5-10), ..., [80-85), 85+ with estimates of age-specific mid-year population counts (L_x)[2]. We estimated the 2019:2018 and 2020:2018 age-specific mortality rate ratios ($RR_{2019,NCHS\text{-}Census}$ and $RR_{2020,NCHS\text{-}Census}$) using 2018, 2019, and 2020 m_x in the NCHS-Census data. To estimate 2019 and 2020 period age-specific death rates, $\boldsymbol{m}_{\boldsymbol{x}_{\boldsymbol{2019}}}$ and $\boldsymbol{m}_{\boldsymbol{x}_{\boldsymbol{2020}}}$, used to generate the 2019 and 2020 U.S. life tables, we multiplied the age-specific $RR_{2019,NCHS\text{-}Census}$ and $RR_{2020,NCHS\text{-}Census}$ with the 2018 period age-specific death rate, m_x, in the official 2018 US life tables:[4]

$$\boldsymbol{m}_{\boldsymbol{x}_{\boldsymbol{2019}}} = m_{2018,Official} * RR_{2019,NCHS-Census} \qquad \boldsymbol{m}_{\boldsymbol{x}_{\boldsymbol{2020}}} = m_{2018,Official} * RR_{2020,NCHS-Census}$$

To generate 2019 and 2020 U.S. life tables, we used $\boldsymbol{m}_{\boldsymbol{x}_{\boldsymbol{2019}}}$ and $\boldsymbol{m}_{\boldsymbol{x}_{\boldsymbol{2020}}}$ and the 2018 a_x in the official 2018 life table[4] to estimate $\boldsymbol{q}_{\boldsymbol{x}_{\boldsymbol{2019}}}$ and $\boldsymbol{q}_{\boldsymbol{x}_{\boldsymbol{2020}}}$. For example, for 2020:

$$\boldsymbol{q}_{\boldsymbol{x}_{\boldsymbol{2020}}} = {(\boldsymbol{m}_{\boldsymbol{x}_{\boldsymbol{2020}}} * n)}\Big/{\left(1 + \left[(n - a_{x_{2018}}) * \boldsymbol{m}_{\boldsymbol{x}_{\boldsymbol{2020}}}\right]\right)}$$

where n is the width of the age interval.

To generate distributions of 2020 life expectancies for each country, we added 10% random uncertainty to $\boldsymbol{q}_{\boldsymbol{x}_{\boldsymbol{2020}}}$ and used Python version 3.9.1 to simulate 50,000 life tables.[5] We report the median (P_{50}) estimate for each life expectancy as well as the fifth (P_5) and ninety-fifth (P_{95}) percentiles as credible ranges for 2020 life expectancies. This was done for the total, male, and female populations of each country, and for the United States, also by race-ethnicity.

Validation Analyses

We validated our analytic approach by comparing our 2019 and 2020 estimates of life expectancies with existing sources. Our 2019 life tables produced 2019 life expectancy estimates that match those reported for the United States by NCHS[6,7] and reported for peer countries in the HMD 2019 life tables. For example, as shown in the **Table** below, our method estimated that female life expectancy in 2019 in Austria and Portugal was 84.13 years and 84.50 years, respectively, aligning with the life expectancies reported in the HMD life tables: 84.19 years and 84.56 years, respectively. Our method estimated that U.S. male and female life

© 2022 Woolf SH et al. *JAMA Network Open.*

expectancies in 2019 were 76.32 years and 81.39 years, respectively, matching NCHS official estimates: 76.3 years and 81.4 years.[6,7]

Second, the 2020 life tables estimated from our method differ little from recently published estimates in the literature and NCHS reports. For example, according to an international study led by Oxford University (Islam et al. 2021[8]), the 95% confidence interval for the decline in female life expectancy that occurred in Austria and Portugal between 2019 and 2020 was 0.51-0.75 years and 0.61-0.94 years, respectively, similar to the "credible range" we report: 0.53-0.83 years and 0.63-0.92 years, respectively. Likewise, among U.S. male and female populations, the 2019-2020 declines in life expectancy estimated by Murphy et al.[9] (2.1 years and 1.5 years, respectively) match our estimates of 2.13 (1.96-2.30) and 1.51 (1.35-1.67) years, respectively.

eTable. Comparison of Published Estimates and Model Predictions for 2019 Life Expectancy and Changes in Life Expectancy Between 2019 and 2020 in the US, Austria, and Portugal

	**Life expectancy (years) in 2019**		**Changes in life expectancy between 2019 and 2020 (years)**	
	**Published estimates**	**Model prediction**	**Published estimates**	**Model prediction**
**United States**				
Males	76.3[6,7]	76.32	-2.1[9]	-2.13 (CR=-1.96 to -2.30)
Females	81.4[6,7]	81.39	-1.5[9]	-1.51 (CR=-1.35 to -1.67)
**Austria**				
Males	79.54[3]	79.58	-0.84 (CI=-0.72 to -0.96)[8]	-0.81(CR=-0.65 to -0.97)
Females	84.19[3]	84.13	-0.62 (CI=-0.51 to -0.75)[8]	-0.68 (CR=-0.53 to -0.83)
**Portugal**				
Males	78.64[3]	78.61	-0.93 (CI=-0.84 to -1.03)[8]	-0.98 (CR=-0.82 to -1.14)
Females	84.56[3]	84.50	-0.78 (CI=-0.61 to -0.94)[8]	-0.78 (CR=-0.63 to -0.92)

CI = 95% CI; *CR* = credible range; *HMD* = Human Mortality Database. Model predictions are estimates produced by the study method. Credible range denotes values at 5th and 95th percentiles from 50,000 simulations. U.S. estimates from the National Center for Health Statistics are reported to only one decimal place.

© 2022 Woolf SH et al. *JAMA Network Open.*

eReferences

1. National Center for Health Statistics. *Restricted-Use Vital Statistics: Detailed Mortality - Limited Geography*, 2018, 2019, and 2020. https://www.cdc.gov/nchs/nvss/nvss-restricted-data.htm

2. US Census Bureau. *Vintage 2020 Estimates, July 1st resident population by state, county, age, sex, single-race, and Hispanic origin.* July 27, 2021. https://wonder.cdc.gov/wonder/help/single-race.html

3. Human Mortality Database. University of California, Berkeley (USA), and Max Planck Institute for Demographic Research (Germany). Accessed January 21, 2022. https://www.mortality.org/

4. Arias, E., and Xu, J. *United States life tables, 2018*. National Vital Statistics Reports; Vol 69, no.12. 2020. Hyattsville, MD: National Center for Health Statistics.

5. Python Software Foundation. Python Language Reference, version 3.9.1. Accessed March 3, 2021. http://www.python.org

6. Arias E, Tejada-Vera B, Ahmad F, Kochanek KD. Provisional life expectancy estimates for 2020. *Vital Statistics Rapid Release 15*, 2021.

7. Kochanek, KD, Xu JQ, Arias E. Mortality in the United States, 2019. *NCHS Data Brief*, no 395. Hyattsville, MD: National Center for Health Statistics. 2020.

8. Islam N, Jdanov DA, Shkolnikov VM, et al. Effects of covid-19 pandemic on life expectancy and premature mortality in 2020: time series analysis in 37 countries *BMJ* 2021;375:e066768

9. Murphy SL, Kochanek KD, Xu JQ, Arias E. Mortality in the United States, 2020. *NCHS Data Brief*, no 427. Hyattsville, MD: National Center for Health Statistics. 2021.

© 2022 Woolf SH et al. *JAMA Network Open.*
